# Factors Related to Diabetes Educator Training and Credentialling to Meet the Needs of Rural and Remote Australians

**DOI:** 10.1111/ajr.70187

**Published:** 2026-04-10

**Authors:** Arjun Chhabra, Andrew D. K. Nguyen, Meredith Hancock, Sandra C. Thompson

**Affiliations:** ^1^ Western Australian Centre for Rural Health University of Western Australia Typically Australia; ^2^ Department of Global Health, School of Health and School of Medicine Georgetown University DC USA

**Keywords:** diabetes management, glucose control, prevention, rural workforce, specialist skills, treatment

## Abstract

**Objective:**

With the burden of diabetes rising in Australia, it is important to understand the challenges for rurally based health practitioners to achieve credentialling as a diabetes educator.

**Setting:**

This is particularly relevant in rural and remote Australia with the shortage of credentialled diabetes educators and the great burden of disease.

**Participants:**

Participants were credentialled diabetes educators (25), health practitioners in the process of credentialling (5), health practitioners who did not complete credentialling (7), and diabetes education academics teaching the qualification required before credentialling (9).

**Design:**

Using qualitative research methods, relevant practitioners involved in credentialling were interviewed. The interviews explored hurdles for rural‐based diabetes educators in achieving credentialling given the required clinical hours and the absence of available local mentors. Interviews were transcribed and thematic analysis followed established methods to ensure research rigour.

**Results:**

Participants described challenges, highlighting an inequitable credentialling process for rural health practitioners. Issues included the inadequacy of material explaining the credentialling process at the time of enrolment, challenges finding mentorship support and gaining sufficient clinical experience for credentialling in a rural context, issues with recording credentialling activities, challenges with distance, and finding available jobs due to the ambiguous scope of practice of credentialled diabetes educators, particularly for allied health professionals.

**Conclusion:**

The findings inform potential improvements for diabetes educator credentialling and offer insights into how processes could be improved to support development of a strengthened rural and remote‐based diabetes educator workforce.

## Abstract (highlights)

**What Is Already Known on This Subject:**

Residents in remote and very remote areas have a higher age‐standardised prevalence rate and were 1.3 times more likely to have diabetes than people in major cities.

Credentialed Diabetes Educators (CDEs) in Australia are specialists who have undergone training to support patients with the highest level of care in the field of diabetes education. There is a shortage of CDEs in rural areas, with consequences for the health and wellbeing of rural residents who have diabetes.

People with diabetes who have access to CDEs have better blood glucose control and reduced complications.

**What This Paper Adds:**

Some rurally based practitioners interested and eligible to become CDEs experienced multiple challenges in obtaining credentialling and maintaining their credentialling.

There was a lack of clarity for many practitioners as they embarked on obtaining additional qualifications in this area. For rural allied health practitioners, there were additional challenges related to scope of practice and settings for practice.

Recommendations which could assist rural candidates to achieve credentialling are made. These include focusing some curriculum in the Graduate Certificate course on rural practice, establishing a dedicated knowledgeable mentor for rurally based candidates seeking credentialling, and improving clarity of the scope of practice of CDEs.

## 1 Introduction

Diabetes mellitus represents a significant public health challenge across Australia, with disproportionately high prevalence and impact in rural and remote communities [1, 2, 3]. Currently, Australia is facing a diabetes epidemic since the population living with diabetes has increased by 32% from 2013 to 2023 to a total of 1.5 million people [3]. Effective prevention, early diagnosis, and multidisciplinary management—particularly involving credentialed diabetes educators—are essential to reducing health disparities. Coordinated efforts by the rural health workforce, professional bodies such as the Australian Diabetes Educators Association (ADEA), and all levels of government are critical to improving outcomes and reducing the long‐term economic and social burden of diabetes nationwide.

People living in remote and very remote areas have the highest age‐standardised prevalence rate and are 1.3 times more likely to have diabetes than people in major cities [1]. Additionally, the rate of death from diabetes was reported in 2020 to be twice as high for those living in remote and very remote regions [4]. In remote areas of Central Australia, 40% of Aboriginal adults were diagnosed with diabetes, among the highest rates of diabetes across the world. The reasons for poor diabetes outcomes in rural and remote Australia are multifactorial, but contributing to this is limited access to appropriate diabetes education for people living in these regions [3].

Increasing diabetes rates nationwide has led to sustained and growing demand for healthcare providers with expertise in diabetes education. To address this demand, the ADEA was established in 1981 to serve as the peak body for diabetes education and management across Australia. It certifies Credentialled Diabetes Educators (CDE) in Australia. The ADEA defines a CDE as a specialist who has undergone training to support patients with the highest level of care in the field of diabetes education [5]. CDEs support people in understanding their diabetes, making informed decisions, promoting self‐care behaviours, and facilitating collaboration across the health care team to improve health status and quality of life [5]. Within the rural health context, CDEs play a vital role in addressing the higher burden of diabetes in these populations and shortages of the local diabetes health workforce [1, 3, 6]. Global research has consistently shown that the involvement of diabetes educators significantly reduces the rate of hospitalisations related to diabetes complications [6]. In Australia, a 2014 ADEA report noted that diabetes education is “highly cost effective”, reducing emergency visits, general practice appointments, and comorbidities [7]. While the outcomes of diabetes educators in clinical care have been studied, research gaps remain regarding the credentialling process itself, particularly in Australia. Moreover, CDEs are particularly important in rural and remote areas which have ongoing issues with the attraction and retention of skilled health professionals which place time pressures on doctors and specialist services. Rural general practitioners are often overstretched and lack the time to provide and reinforce education so value expert allied health support [8].

The ADEA recognises eligible health professionals (registered nurses, midwives, dietitians, medical practitioners, pharmacists, podiatrists, exercise physiologists, physiotherapists and optometrists) as qualified to undertake the diabetes education credentialling process [5, 9].

Credentialing is appealing to those healthcare providers seeking to expand their scope of practice, as it provides formal recognition of their expertise, enables them to authorise registrations for the National Diabetes Service Scheme and to access Medicare and other billing pathways, thereby enhancing the quality and affordability of care for patients [7]. Recognition by Medicare and the Department of Veterans' Affairs, as well as some private health insurers, is an advantage for those health professionals who otherwise cannot currently provide subsidised billing options for diabetes education. At the time of data collection for this study, the credentialling process consisted of a Graduate Certificate in Diabetes Education and Management, 1000 h of clinical experience, a six‐month mentorship experience to achieve credentialling, an ongoing requirement for 20 h of continuing professional development (CPD) per year under ADEA supervision, and a referee report [5]. These requirements were updated in late 2024 (while this study was underway) with the most notable changes being that the required supervised clinical hours decreased from 1000 to 500 and the introduction of micro‐credentialling opportunities was foreshadowed [5, 7].

At the time of undertaking the research, there were eight Australian institutions that award this Graduate Certificate (Table 1). Gaining insight into experiences with the process is essential for enhancing CDE credentialling outcomes in rural and remote communities, thereby supporting efforts to address the increasing burden of diabetes credentialling.

**TABLE 1:** Information detailing Graduate Certificate Courses in Diabetes Education.

Institution	Location	Course Length	Delivery	Placement Length	Commonwealth Support Status
Deakin University	Melbourne, Victoria	12 months	Flexible, one tutorial in‐person	1 week	No
Curtin University	Perth, Western Australia	6 months	Online	1–2 weeks	Yes
University of Technology, Sydney	Sydney, New South Wales	12 months	Online	1 week	Partial
James Cook University	Townsville, Queensland	8 months	Online w/3 day residential	1 week	Yes
Southern Cross University	Gold Coast, Queensland	8–12 months	Online	1 week	Yes
Mayfield Education	Melbourne, Victoria	12 months	Blended	1 week	No
Flinders University	Adelaide, South Australia	12–24 months	Online	1 week	Yes
Western Sydney University	Sydney, New South Wales	24 months	Online	1 week	No

This study aimed to explore the specific experiences and needs of rural practitioners related to credentialling given the importance of access to CDEs for improving diabetes care for rural residents in Australia.

## 2 Methods

**FIGURE 1:**
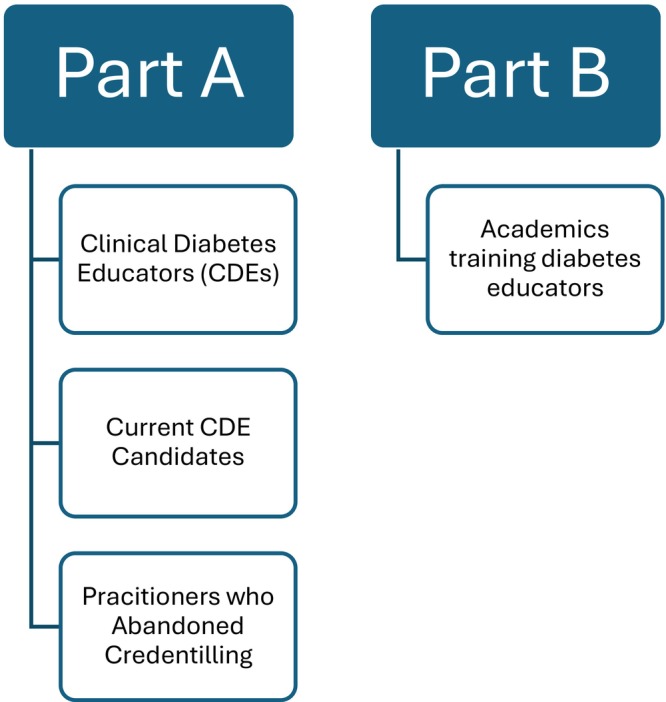
Breakdown of participants interviewed into Part A and B.

The study was approved by the University of Western Australia Human Research Ethics Office on 19 August 2024 (2024/ET000614). This study was conducted by interviewing two groups (Figure 1). Part A entailed interviews to understand the experience of the credentialling process and maintaining CDE accreditation from the perspective of CDEs and exploring the experience of enrolled CDE candidates and health practitioners who had abandoned credentialling, with a particular focus on rural and remote‐based candidates. Part B was interviews with Diabetes Educator Graduate Certificate academics to understand their perspectives of rurality in their courses. All four authors (2 male and 2 female) undertook interviews; three had formal training in qualitative research and one was trained specifically for this project. Three were academics and one was a pre‐med global health student. There was no preexisting relationship between the interviewer and the research participant with three exceptions. One was that one investigator had indirect knowledge of the delays in credentialling experienced by a remotely based allied health professional who was a relative of a work colleague; the indirectly reported experience of this person had raised the researchers' awareness of this issue and underpinned the initiation of this study. A second participant was no longer rurally based but was known to two of the research team through her previous work in the rural region in which they were based and was known also to have had challenges achieving the hours for credentialling; neither researcher was involved in her interview. A third CDE participant was known to the CDE member of the research team through professional networks but was not a close colleague.

For recruitment (both Part A and B), a participant information sheet containing information about the study and consent form was sent by email from the Western Australian Centre for Rural Health (WACRH) researchers to diabetes educators, universities, healthcare networks, and other related professional organisations. This was sometimes preceded by telephone or video (Teams) contact. After each interview, participants were asked to reach out to colleagues to invite their participation in the study. For Part B, the academics from the eight higher education centres offering a course in Diabetes Education were invited to participate.

The interview schedules (Supplementary File 1) with questions and follow‐up prompts if needed were developed within the research and reviewed by colleagues. It covered demographic information, experience with the credentialling process, and suggestions for improvements to the process. A second interview guide for academics (Supplementary File 2) covered information regarding the course which they taught and any suggestions they had for improving the credentialling process for rural students. All participants provided informed consent to the study; the initial part of the interview via Microsoft Teams explained more about the study and researcher. Individual interviews ranging from 30 to 60 min were recorded. Transcripts were autogenerated via Teams and checked against the recordings and corrected for accuracy. Participants were offered the opportunity to edit their transcript and to clarify their meaning before data entry onto secure study servers and before data analysis. While based on the interview schedules, the focus of interviews varied depending on the experience of the interviewee and the topics they chose to highlight. The research team regularly debriefed throughout the process of data collection and analysis to clarify information, increase recruitment and ensure themes were robust.

Our analysis was guided by fundamental qualitative description [10] using an inductive analysis approach to identify themes related to potential barriers and facilitators for meeting the requirements for diabetes educator credentialling and the maintenance of CPD. This occurred through multiple readings and comparison of the interview transcripts by members of the research team (AC, AN, MH). The themes found were discussed and compared in meetings within the research team to verify findings, clarify concepts, increase research rigour, and reduce bias by ensuring the full range of responses were considered [11]. Quotes that supported these themes were thematically organised as per the coding tree developed by the research team (Supplementary File 3) with regular discussion and debriefing among the team on emerging findings.

## 3 Results

From August to November 2024, 25 CDEs, 5 CDE candidates, 7 health practitioners who abandoned credentialling, and 9 academics were interviewed. Among the interviewees from Part A, there was a professional breakdown of 17 accredited practising dietitians, 18 registered nurses, and 1 pharmacist, whereas Part B consisted of the 9 academics. A total of 3 males and 34 females were included in the study. Interviewees were based in every major state and territory except for the Australian Capital Territory, in population centres from less than 100 to 200 000 people. Participants' Modified Monash Model (MMM) ratings ranged from 2 to 7, with 9 in regional centres (MMM 2), 22 in rural centres (MMM 3–5), and 11 in remote or very remote locations (MMM 6–7).

Participants described multiple challenges, with the most significant and recurring issues summarised in the themes: the lack of Graduate Certificate course material focused on the credentialling process and rural‐based diabetes education, difficulty finding a local mentor, obstacles with credentialling as a rural and remote‐based candidate, struggles for job opportunities for those in rural locations and from allied health backgrounds, lack of clarity and detail around the new micro‐credentialling offerings, complaints about the logging process for rural‐based educators, and challenges with distance.

### 3.1 Lack of Graduate Certificate Course Material Explaining the Credentialling Process and Being a Rural‐Based Diabetes Educator

Participants commented that when beginning the course, they had lacked understanding of what the credentialling process entailed including the clinical hours and mentorship process required. The course coordinators stated they often provide a brief overview of credentialling and refer the students to the ADEA website for more information. However, many waited until the latter half of the course to go into depth regarding the process, noting that not all students plan on becoming credentialled, so they do not want to bombard them with information. Others stated they did not want to provide the students with the wrong information as their knowledge of the credentialling requirements could be out‐of‐date. Part A participants reported they would have preferred a full understanding of the credentialling process before the course, as this could have saved their time and effort including by allowing them to log their clinical hours during the Graduate Certificate course.

In their respective Graduate Certificate curriculums, many CDEs did not remember a particular focus on rural and remote‐based diabetes care. A rural CDE stated: “You know, obviously low SES [socioeconomic status], not a lot of money, you know very poor health literacy – those things weren't explained to us in that course. I just knew about it because I was already in the area” *(Participant 1, CDE)*.

Some of the academics recognised that there was not a particular rural focus, highlighting that there was inadequate time to add additional course material: “I don't think there is a specific focus on rural. No, no, I can't think of anything specifically. Look I think the course is so jam packed with content, it's hard to see how we'd add any more to it” *(Participant 33, Academic)*. Other academics included a focus on rural diabetes education, as one stated:

“We have two choices, so one focuses on working with Aboriginal Torres Strait Islander people where we have an Aboriginal CDE come in and talk about that and their experience working rurally and remote. And we also have a GP [general practitioner] come in and talk about working in a rural remote area, talking about working with culturally and linguistically diverse populations.” (Participant 38, Academic)

After hearing about working in rural and remote areas, a CDE mentioned they were inspired by a guest lecturer to work in a rural setting: “Yeah, I did have [a guest lecturer] in my postgrad course. I had one woman … and she had been out remote WA [Western Australia], and she shared her experiences with us in the group and that's what actually inspired me to go out myself. So, if one person was to share their experiences about working in one of these remote areas that that could inspire one more person” (Participant 15, CDE).

### 3.2 Difficulty Finding a Local Mentor to Provide Consistent and Relevant Support

To achieve credentialing, a candidate must complete a 6‐month mentoring process in which the mentor and mentee meet for at least an hour each month. A common theme of mentees was that their mentor could not or did not properly support them throughout the process. As one rural CDE stated:

“I think I had a couple of phone calls with her and then I had nothing, so I didn't really have a mentor. That was really, really hard. You had no one to bounce ideas off. No one else in town does the job, so no one else could advise you. You have no idea who else in the area is credentialled that you could chat.” (Participant 10, CDE)

Rural and remote‐based candidates often had trouble finding a mentor and when they did, it was likely someone who could only mentor virtually. Interviewees reported lacking anyone to consult with questions; moreover, mentors often could not address issues specific to rural‐based care. One rural‐based CDE spoke about the benefit of having a local mentor in a rural setting: “Nothing really prepares you for sometimes the confronting things that you see, but to have somebody you can actually debrief with … someone that understands the locality” (Participant 15, CDE). Many emphasised the need for a more standardised mentor selection process to ensure mentors had the knowledge and time to provide sufficient support. Participants suggested enhancing the ADEA portal's mentor finding platform to enable two‐way communication, enabling mentees and potential mentors to discuss focus areas and regions for more effective matches.

An example of strong institutional support leading to greater numbers of rural CDEs was highlighted in interviews with South Australian (SA) participants, where recent graduates would be linked with the South Australian Rural Support Service (RSS) including an orientation package of resources, individualised development plans codeveloped with unit managers, regular network meetings and CPD workshops with supported travel to Adelaide. One CDE attested:

“So, the managers where I work are very supportive even though they know nothing about diabetes. … we have a regional training each year for two days … (the SA RASS organiser) met me there and she said to me, she said, look, if you need a mentor, I'm happy to do it. So I took her up on it and I've come quite good friends with her.”(Participant 25, CDE)

### 3.3 Challenges and Opportunities With Credentialling and Clinical Experience as a Rurally‐Based Candidate

Interviewees from rural and remote settings emphasised their difficulty in credentialling. Many stated it was tough to get clinical hours in rural and remote communities because the caseload is less and more sporadic. One CDE who nearly completely abandoned credentialling described her struggles:

“See people you know with diabetes … would sometimes not be in the community or they didn't want to come for their appointments because of other difficulties in their lives and other things going on so to get the 1000 hours was very difficult. So, I left my credentialling‐I thought, well, it's all too hard.” (Participant 4, CDE)

It was stated that with working in remote communities, travel was a barrier and often virtual diabetes education was not an option. When telehealth was available, there were many difficulties including internet access and having the patient present for meetings as scheduled. CDEs who had this experience believed that ADEA's new adjustment to only requiring 500 h of clinical experience would be helpful to current and future candidates. Many participants noted a regional shortage of diabetes educators, a clinician undersupply, or challenges finding job opportunities to accrue clinical hours. Multiple interviewees mentioned their workplaces had vacancies in diabetes educator positions for months at a time. Many rural and remote CDEs reported heavier workloads and limited formal or informal mentoring, as they may be the sole CDEs in their area and surrounding towns. In more remote Aboriginal communities, additional cultural training was often learnt on the job, with participants suggesting its inclusion in ADEA's new micro‐credentials. Some CDE candidates lacked a formal local mentor, and many in rural and remote communities had no colleagues or reference points when starting a new role. A rurally‐based CDE and academic commented:

“… they often had to go into a job with no one to really learn the job from. And they hadn't done the course because they're in country and they needed to fill positions, so they had to just sort of learn on the job and … get the clinical exposure and the breadth of exposure. It can be really hard when you're in a rural service that doesn't have the allied health workers and endocrinologist.” (Participant 40, Academic)

Several rural‐based CDEs requested an ADEA liaison to respond promptly to queries, mentor rurally trained CDEs and enhance the credentialling and clinical experience in rural and remote regions. CDEs nationwide described various incentives that had effectively recruited and supported rural and remote diabetes educators. One approach involving hiring CDE candidates to gain clinical hours while working at an institution, with the expectation they remain employed after credentialling.

### 3.4 Job Opportunities and Career Advancement for Diabetes Educators in Rural Locations

Although CDE credentialling is open to nurses and various allied health professionals, allied health candidates more frequently reported difficulties finding rural employment. CDEs with allied health backgrounds noted that a key advantage of becoming a CDE is the opportunity to bill for diabetes services through Medicare, Australia's universal health insurance scheme. Many allied health professionals felt workplaces expected nursing skills for these roles, limiting their ability to apply for or secure roles despite holding CDE qualifications:

“I work in the hospital, but I do share an office space with our diabetes educators and they're finding it so hard to find staff. They're constantly putting out expressions of interest for people, but they're always nurse positions advertised as nurse diabetes educator positions. And so, you know, other people don't feel like they can apply for those positions.” (Participant 36, Dietitian)

Multiple CDEs reported that their job descriptions and pay remained unchanged, despite enhanced CDE skills. Participants indicated that, should they leave their current positions, their successors would likely not be required to hold diabetes education qualifications, thereby risking a loss of essential CDE support for the community. The prevailing lack of clarity regarding their scope of practice affected compensation and job opportunities, particularly for allied health professionals. Some CDEs wanted clarity as to whether the scope of practice differed based on professional discipline, a clarification considered particularly important in rural practice:

“… it's not clear from an ADEA perspective, the scope of practice of a CDE nurse compared to a CDE dietitian compared to a CDE pharmacist compared to a CDE podiatrist for example and so forth. And I think it's really important that ADEA is transparent with regards to what particular professions can and cannot do, because…in country, we need people to be generalist specialists to a degree and we need them to be able to cover a multitude of cohorts and patient settings, aged care, acute community, paediatrics, obstetrics. And so, we need to employ people that obviously can provide us with the skills that are required in this space.” (Participant 25, CDE)

Financial issues were frequently raised, including participants suggesting that people may be more likely to complete their credentialling if there was better financial support:

“Financially that initiative might be there for people if there's more clarity from ADEA based on, you know, if you have different levels as credential diabetes educator and if you do hold a job that has this position description that covers what you do, you're seen as a senior practitioner” (Participant 35, Dietitian).

### 3.5 Lack of Information Around the New Micro‐Credentialling Offerings

The ADEA has recently introduced micro‐credentialling for CDEs and CDE candidates, targeting specific skills for credentialling or CPD requirements. These opportunities were being promoted as a cost‐effective, flexible way for CDEs to stay current with evidence and earn badges to enhance their resumes [5]. The perspective among many CDEs interviewed was positive towards virtual refresher training such as on insulin pump usage, but they also expressed confusion on the overall goal of micro‐credentials. The additional cost of micro‐credentials was noted as a barrier, and others questioned whether completing them would alter their scope of practice. Interviewees questioned the validity of course assessments, suggesting that supervisors in each workplace could evaluate and sign off on these skills. Despite concerns, many indicated they would sign up for offerings that would allow them to stay up to date despite the rural location of their practice.

“I love the idea of the micro‐credentialling that ADEA are putting in … there's going to be big parts of diabetes management self‐education that you may not get exposed to depending on, especially for us in those remote and rural areas. Very different obviously where compared to those big metro centres where you just have lots of people, a big team and you know lots of doctors. A lot of us are working on our own.” (Participant 7, CDE)

There was a considerable range of perspectives to the idea of micro‐credentialling, from great caution to enthusiasm. Some rural‐based participants commented that they would like to see more continuing professional development (CPD) from the ADEA with rural and allied health perspectives.

### 3.6 Rural Concerns Related to the Logging Process During Credentialling and for CPD

Logging into the ADEA system was widely described as clunky and time‐consuming. This was particularly problematic in rural and remote areas with unreliable internet, with one interviewee emphasising that they went into the office for hours on their day off to log requirements: “I found the online system clunky and awful. It was just so hard to use, to upload documents, to keep a track of everything, even just logging in, logging out was a pain” (Participant 5, CDE). To improve this process, some suggested that the ADEA could adopt a method used in other fields in which photographs of the logs could be taken and uploaded. Additionally, participants were unclear about certain requirements, such as which logged credits and CPD activities required ADEA endorsement. There was also uncertainty regarding the benefits of full versus student ADEA membership, including whether student members could log their clinical hours.

### 3.7 Challenges With Distance, Cultural and Telehealth Considerations in Rural and Remote Locations

Participants described many challenges of undertaking their credentialling from a rural setting which often related to the work pressures on practitioners in those settings. Some examples of these difficulties are evident in the sentiments below.

“I guess some acknowledgement of the fact that when you work in in a rural setting, you're quite often a generalist rather than a specialist, and so the fact that those hours count towards your credentialing as a diabetes educator may be as a part of a generalist workload as well as specific diabetes work” (Participant 35, CDE).

Many comments highlighted that those working in CDE pathways roles were aware of their isolation and wanted support that understood their rural context. These wishes extended across support during training to covering other professional concerns in practice.

“Leaving your workplace when you're the only person covering your role in the hospital and then feeling like your workload isn't covered there because you're the only person there for that day… That leaves a gap in the service. Whereas I guess if you were at a bigger location, for those more urgent things there would be someone on hand in the department” (Participant 36, Dietitian)

## 4 Discussion

The research sits within a much larger body of scholarship that has recognised the recalcitrant nature of addressing rural health workforce shortages and presented approaches that can guide efforts to improve attraction and retention of health workers with relevant skills, and that multiple strategies are needed in terms of training, incentives and place‐based support [12, 13, 14]. Our study was narrowly focussed to specifically look at credentialling of diabetes educators and took place during a time of credentialling changes in Australia, which made it difficult to collaborate with the ADEA in designing and implementing this research. The credentialling changes were announced at the Australasian Diabetes Congress in August 2024 but were not available online for weeks, highlighting the challenges particularly for rural and remote CDEs to be informed and the inequities in access to information. However, the timing allowed participants to respond to the proposed changes to the researchers while discussing the opportunities and challenges they had experienced with the credentialling process.

The interviews highlighted that increasing remoteness increased the difficulty of the credentialling experience for a CDE. Adjustments to the credentialling process are needed to address some of the extra challenges for rural candidates, over and above improving and streamlining processes for all CDE candidates. Some requested changes are straightforward, such as providing greater transparency about the credentialling process before enrolment and having course coordinators explain requirements to students at the start of the Graduate Certificate course. While students had a general sense of the credentialling process, many were unaware of the time commitment, details of the process and of the different ADEA membership levels. An in‐depth review of the credentialling process would give potential CDE candidates time to consider it, begin logging hours during the Graduate Certificate course, and increase the likelihood of completing the CDE requirements.

Given rural and remote‐based students' uptake of online courses, a greater emphasis is needed on supporting development of skills relevant to rural and remote settings. With telehealth and fly in‐fly out work (FIFO) commonly used to service rural areas of Australia, metropolitan‐based CDEs would also benefit from learning this skill for providing services to people living in rural or remote communities [15, 16]. Increasingly there is recognition that to achieve an effective rural health workforce and focus on rural health equity, ruralisation is needed across all health professional education [17]. As highlighted by our participants, relevant skills must include understanding social determinants in a rural health context and the unique challenges for patient self‐management, an issue identified in the broader rural workforce literature [12]. This suggests that for those that will work in a rural setting, factors such as health literacy and socioeconomic status may be useful topics to highlight in the Graduate Certificate course. In the Graduate Certificate course, modules and guest speakers addressing characteristics of rural practice could better prepare students for the variety of work encountered in rural settings. Seeing and hearing from rurally‐based practitioners during their course could increase interest and attract rural‐based diabetes educators in the future [18]. Efforts to upskill the broader CDE workforce must be balanced with addressing the connectivity and service disruptions commonly experienced by rural CDEs. These include interruptions to internet, phone, and transport services. Living costs are also disproportionately higher in rural and remote areas, impacting both patients and healthcare providers. Addressing these challenges should include enhanced financial, family support, and tax incentives to encourage health professionals to reside in rural locations. Measures such as the Commonwealth Prac Payment for teaching, social work, nursing, and midwifery students could be specifically implemented focussing on rural diabetes educator candidates [19, 20].

Some participants suggested that mentors complete standardised training to enhance the mentorship process for both mentor and mentee. As positive mentor‐mentee relationships are associated with better credentialling experiences, careful matching is especially important in rural settings. Consistent, relevant guidance from mentors has been shown to effectively support career development [21]. Many rural and remote candidates lacked a local mentor or someone that could support them with the challenges they faced on a day‐to‐day basis and felt isolated and inadequately supported. Therefore, alongside enabling CDE candidates to connect with potential mentors via the current platform, appointing an ADEA liaison for rural and remote candidates could help identify CDEs with relevant experience in a similar setting. An example is a system modelled on the South Australian Rural Support Service (SA RSS) which supports various fields of rural practitioners, including diabetes educators with orientation resources, continued training and quality assurance checks. This program is funded by the state government and successfully supports the needs of these educators in a rural context [22]. Thus, a similar rural support network, or rural‐focused liaison in the ADEA could increase the rate of those who complete credentialling in those regions and, in turn, incentivise more rural health practitioners to credential. Having rural representatives on all ADEA state branches and networks may also help ensure that all decisions and programming consider rural candidate and CDE practitioner needs. There is also potential to establish and evaluate rural and remote CDE communities of practice. This approach has worked for some other rural practitioners [23, 24], but without resourcing and a committed facilitator achieving the value creation described by Wenger and colleagues may not be achieved [25, 26]. However, activities, interactions and building of knowledge capital could lead to opportunities for future higher order changes in practice and improvements in performance.

Some of those interviewed felt that it was easy to gain clinical hours if their role was specific to diabetes education, but this was less likely for rurally based practitioners and practitioners from allied health backgrounds. Rural CDE candidates faced challenges such as a less consistent patient load, whether it was due to distance or struggles with telehealth. ADEA's recent change to requiring 500 rather than 1000 h of clinical experience was welcomed as it makes it easier for rural and remote‐based practitioners with inconsistent diabetes‐specific caseloads, a positive step towards more equitable credentialling.

For transparency, the importance and scope of practice of credentialed diabetes educators should be available and publicised. Despite the information available online, some participants reported that many employers even in rural areas have not embraced diabetes educators from allied health backgrounds such as dietitians and exercise physiologists for employment as CDEs. A 2025 study found that Graduate Certificate students from allied‐health backgrounds had trouble finding a placement organisation [27]. Arguably, the ADEA needs to do more in promoting the skills of allied health practitioners in diabetes education given the commonly reported preference for employing nurses is discouraging other practitioners from completing the credentialling process. In 2022, the ADEA published a description of the scope of practice of CDEs in Australia which explains, in certain states, diabetes educators from nursing backgrounds can initiate insulin therapy whereas diabetes educators with allied‐health backgrounds cannot [9]. The issue of whether there are restrictions on CDEs from allied health backgrounds initiating insulin warrants more attention, along with any other differences in scope of practice in managing diabetic care. Discussions must acknowledge the issues and complexities of defining scopes of practice due to the evolution of professions and the wider, more generalist nature of rural health professional work [28, 29]. Based on these authors' examination of these issues, it has been proposed that tasks (competencies) should be regulated as opposed to professions, with rewards and indemnity matched to the level of skill and risk required to perform tasks rather than a professional title.

Throughout Australia, job opportunities for diabetes educators are insufficient, particularly in rural and remote regions. A lack of job opportunities for CDEs and non‐credentialled diabetes educators disincentivises them from completing credentialling [27]. With a clear scope of practice from the ADEA, having a credentialled diabetes educator regardless of professional background would be preferable to leaving a position unfilled for an extended period, particularly in rural and remote areas. Overall, ensuring the scope of practice for all CDEs is known and utilising their expertise is urgently needed to support optimal diabetes care in Australia, so removing barriers to employment of CDEs of all backgrounds warrants attention.

The introduction of micro‐credentials may further obscure the boundaries of professional scope of practice. Of note, there have been mixed assessments of the utility of micro‐credentials with criticisms related to lack of pedagogical theories, limited focus on higher‐level competency assessment and no clear evidence of their distinct value as a teaching modality [30, 31]. Multiple diabetes educators mentioned a need for clarity if their scope of practice changes with completing micro‐credential opportunities. Participants' reception to the concept of the new micro‐credentialling opportunities was mixed, with concerns related to lack of detail on what they are as well as the costs; some interviewees saw them as potential opportunities to refresh their knowledge and skills while others wanted clarity on the benefits for the costs incurred. The potential for micro‐credentials to focus on rural and allied health subjects could also benefit those not working in traditional metro‐centric and nurse‐based practice. More experience and evidence on the value of micro‐credentialling is needed.

Based on the themes identified, several recommendations (Table 2) are made relevant to ADEA, Graduate Certificate Course Coordinators, and other stakeholders. The recommended solutions are likely applicable to support more equitable processes for rural and remote‐based health practitioners globally.

**TABLE 2:** Recommendations to Relevant Stakeholders.

Findings	Recommendations	Rationale
Graduate Certificate students felt a lack of understanding of the credentialling process	The ADEA should provide course coordinators with a complete presentation explaining the credentialling process, which describes opportunities to log hours while candidates are still completing their academic training, as well as the eligibility and benefits of Associate and Full membership of ADEA Course coordinators should explain the credentialling process at course orientation, including students who are eligible to log placement hours	Graduate Certificate students are often not aware of the complete credentialling process during the course which can become a hurdle to credentialling By providing course coordinators with this presentation, they would share the most up‐to‐date information about the credentialling process with their students
CDE candidates, particularly those rurally based, discussed the difficulty in finding a well‐suited mentor	The ADEA should update the mentor‐finding platform to allow for two‐way communication and ensure mentors have rigorous standardised training	This could allow for mentors to be better suited for the needs of their mentee
Many participants mentioned the difficult in logging clinical hours for credentialling and CPD	The ADEA should adopt a method used in other fields in which photographs of the logs could be taken and uploaded	The logging process needs streamlining because it is consistently time‐consuming and frustrating
Rural and remote‐based CDEs often described a lack of support network	The ADEA should provide a liaison as an available resource to ADEA members living in rural and remote areas, and provide advocacy representation for rural‐based CDEs	This will benefit rural and remote‐based CDEs who often lack local support and have questions about practice and scope advocacy
Participants indicated other healthcare provided were unaware of the role of CDEs	The ADEA and other relevant stakeholders should advocate and publicise the importance of CDEs, and define a CDE's role	The scope of practice of CDEs and CDE candidates is currently unclear to employers
CDE candidates and CDEs, particularly those from allied‐health fields, discussed difficulty finding job opportunities	Define scope of practice and highlight a regularly updated jobs board	Clarification can lead to job opportunities and increased pay, particularly rurally while updated jobs boards can provide regular opportunities for CDE candidates
Participants described a lack of material in the course focused on rural‐based healthcare	Some courses invite in guest speakers and have modules focused on Aboriginal and rural health; this approach to curriculum and student learning should be adopted by all courses	This would help prepare rural and remote‐based CDE candidates, potentially inspire more people to work in rural and remote settings and would generally increase understanding of the care of people with diabetes who reside in rural and remote areas

This qualitative study ensured rigour by being conducted in a systematic, thorough, and accurate way. Attention was paid to the design of the study, the sampling plan, the data collection methods, and the data analysis methods. We utilised multiple approaches to recruitment and triangulated findings from different categories of participants. However, there were some limitations. While the study aimed to capture perspectives from enrolled Graduate Certificate students, recruiting them proved difficult due to challenges with access to them and our approach for participation in interviews occurring late in the semester during assessment periods. Thus, the interviews reflected CDE graduates' experiences with courses rather than capturing the current experiences of students in the Graduate Certificate courses. Some courses may have already changed. However, given multiple interviewees completed their Graduate Certificates in recent years, it is unlikely that the themes would be very different. The ADEA is in the process of changing their credentialling approach, but there were deficiencies in communicating these intentions in advance. Also of note, after data collection was completed, another institution began offering a Graduate Certificate course and the ADEA added further requirements to become a CDE, including completing the Professional Practice Micro‐credential and a practical assessment [5].

## 5 Conclusion

The high rates of diabetes and documented poorer outcomes in morbidity and mortality in rural Australia require that workforce shortages for diabetes care are addressed. This research has highlighted several issues experienced by CDEs and CDE candidates relevant for rural employment and practice and has captured suggestions for approaches by which the inequities in workforce capacity could be addressed. The recommendations provided have a particular focus on supporting the rural workforce but can have benefits for all CDEs and CDE candidates throughout Australia as well as for similar credentialling processes around the world. Future research could gain insights from a larger sample of Graduate Certificate students to better understand successes and challenges within the course and examine the content of course materials and experience with micro‐credentials for rural CDEs and students.

## Author Contributions

**Arjun Chhabra:** conceptualisation, data curation, investigation, formal analysis (lead), methodology, validation, writing – original draft preparation. **Andrew D. K. Nguyen:** conceptualisation (lead), data curation, investigation, formal analysis, methodology, project administration, validation, writing – review and editing. **Meredith Hancock:** data curation, investigation, formal analysis, methodology, validation, writing – review and editing. **Sandra C. Thompson:** conceptualisation, data curation, analysis, methodology, supervision, validation, writing – review and editing.

## Disclosure

This research did not receive external funding. AN, MH, ST were all employed by the WA Centre for Rural Health, University of Western Australia, which is funded under the Rural Health Multidisciplinary Training program of the Department of Health, Disability and Ageing. Meredith Hancock is a Credentialed Diabetes Educator and a Member of the Australian Diabetes Education Association.

## Ethics Statement

The study was approved by the University of Western Australia Human Research Ethics Office on 19 August 2024 (2024/ET000614).

## Conflicts of Interest

The authors declare no conflicts of interest.

## Supporting information


**Supplementary File 1** Interview Guide for Part A: Credentialed Diabetes Educator, Students undertaking their Graduate Certificate course, and Graduates of the Graduate Certificate coursework who abandoned credentialling.

**Supplementary File 2** Interview Guide for Academics teaching in the Graduate Certificate courses.

**Supplementary File 3** Coding tree developed by the research team to systematically capture emerging findings.

## Data Availability

The data that support the findings of this study are available on request from the corresponding author. The data are not publicly available due to privacy or ethical restrictions.
