# Supplementary material for: Factors Related to Diabetes Educator Training and Credentialling to Meet the Needs of Rural and Remote Australians
Source: Aust J Rural Health. 2026 Apr 10;34(2):e70187. doi: 10.1111/ajr.70187 (PMC13069227; doi:10.1111/ajr.70187)
Supplement: Supplementary file 1 — Interview Guide for Part A: Credentialed Diabetes Educator, Students undertaking their Graduate Certificate course, and Graduates of the Graduate Certificate coursework who abandoned credentialling. [file AJR-34-0-s002.pdf]

Supplementary File 1: Interview Guide for Participants in Part A, Clinical Diabetes Educators

Diabetes Educator Interview Schedule

Thank you for consenting to the interview and reading the participant information sheet. You are eligible to participate in this research study given your background in diabetes education. As your interviewer, I will be asking a series of exploratory questions, which I will ask for your response.

Firstly, I'd like to find out a bit more about you and your experiences in diabetes education:

- What is your age?
- What is your gender?
- What is your current occupation and role within the field of diabetes education?
- What was/is your eligible primary health discipline before commencing diabetes educator credentialling?
- Where state and local area do you live in?
    - How long have you lived in this location?
- What state and local area do you work in/have your placement in?
    - How long have you practiced in this location?
- What is the approximate population size of your practice location?
- What is the approximate size of the area you cover with your services?
- When did you graduate from your Graduate Certificate in the field of Diabetes Education?

Can you please talk about why and when you got interested in being a diabetes educator and your pathway to where you are now, including any challenges you have experienced along the way.

Take your time answering the questions from the following questions, we would appreciate your thoughts and explore the reasons for your responses based on the following questions:

- What are your thoughts on whether there is a shortage of diabetes educators in your area?
- What are your thoughts on diabetes educator provision with rural and remote areas compared to metropolitan areas?
- If you are a current diabetes educator candidate:
    - What is your closeness to completing diabetes educator credentialling?
    - What are your remaining requirements left to obtain credentialling as a rural or remote based educator?
- If credentialed as a diabetes educator: How are Continuing Professional Development (CPD) requirements maintained?

    - Which field?: Category 1: Clinical Practice/Diabetes Education/Counselling, Category 2: Management/Administration/Leadership, Category 3: Research, Category 4: Professional contribution)
- If you are a diabetes educator candidate who has abandoned training:
    - Can you provide an explanation of reasons why?
    - What suggestions do you have for improvement?
- What is your awareness of the requirements to be credentialed as a diabetes educator while completing your graduate certificate?
    - Do you have any communication/education from the University?
    - What is the engagement from Australasian Diabetes Educators Association?
- What are your thoughts on whether diabetes educator credentialling and or/maintaining CPD as a diabetes educator has helped your practice as a rural health professional?
    - In what way?
- What are the challenges you have faced in achieving credentialling as a diabetes educator candidate or maintaining CPD as a diabetes educator?
- What are your thoughts and experience on whether being based in a rural or remote area has added to your challenges meeting credentialling requirements or maintaining CPD requirements?
    - In what way?
- What are your thoughts of the ADEA proposals on micro-credentialling
    - How might this affect your credentialling or maintenance of CPD requirements?
- What suggestion(s) do you have to improve the process of diabetes educator credentialling/maintaining CPD for rural and remote based candidates?

This concludes the interview. Do you wish to review and edit the associated transcript for this interview?

All responses will be confidential and used only for the purposes of this study.
