# Supplementary material for: Factors Related to Diabetes Educator Training and Credentialling to Meet the Needs of Rural and Remote Australians
Source: Aust J Rural Health. 2026 Apr 10;34(2):e70187. doi: 10.1111/ajr.70187 (PMC13069227; doi:10.1111/ajr.70187)
Supplement: Supplementary file 2 — Interview Guide for Academics teaching in the Graduate Certificate courses. [file AJR-34-0-s003.pdf]

Supplementary File 2: Interview Guide for Participants in Part B, Academics teaching in Graduate Certificate of Diabetes Education Courses

Higher Education Provider Interview Schedule

Thank you for consenting to the interview and reading the participant information sheet. The research team is aware of several rural-based diabetes educator candidates struggle to receive accreditation. You are eligible to participate in this research study given your background and/or academic role in diabetes education. As your interviewer, I will be asking a series of exploratory questions, which I will ask for your response. Take your time answering these, we would appreciate your thoughts and explore the reasons for your responses based on the following questions:

Questions

- Can you please tell us the proportion of each annual cohort from the last 10 years, whether they are from rural and remote areas?
- Can you please tell us the proportions from each cohort that has gone on to complete credentialling?
- Can you please detail if the credentialling requirements and pathway are discussed with students in the Graduate Certificate?
- Can you please describe if there is any rural focus in the teaching of diabetes education at your institution?
- Can you please provide the estimated costs associated with completing the Graduate Certificate at your institution?
- Are you aware of any ongoing follow up (if any) between the qualification/university and graduate?
- Can you please describe any ongoing links between the qualification and past graduate, especially rural students?
- Can you describe in your own words, any reports of challenges of graduates in obtaining credentialling, especially rural/remote based students (i.e. outside metropolitan areas)?
- Do you have any suggestions to improve the process of becoming a diabetes educator?

This concludes the interview. Do you wish to review and edit the associated transcript for this interview?

All responses will be confidential and used only for the purposes of this study.
