# Supplementary figures and images for: Factors Related to Diabetes Educator Training and Credentialling to Meet the Needs of Rural and Remote Australians
Source: Aust J Rural Health. 2026 Apr 10;34(2):e70187. doi: 10.1111/ajr.70187 (PMC13069227; doi:10.1111/ajr.70187)

**Supplementary File 3: Analysis Coding Tree**

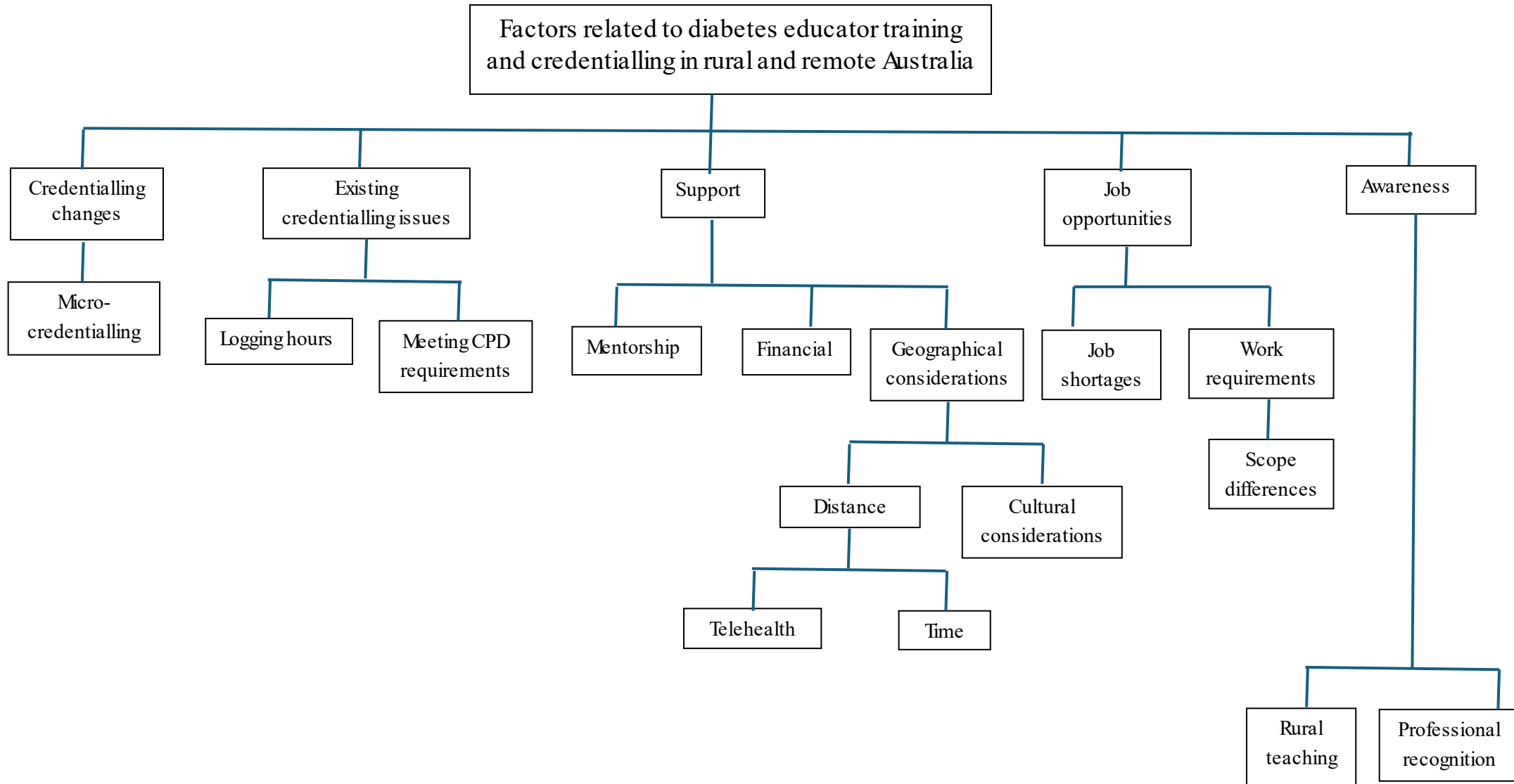

Supplement: Supplementary file 3 — Coding tree developed by the research team to systematically capture emerging findings. [file AJR-34-0-s001.pdf]
